# Needlestick and Sharp Injury Among Dental Instrument Reprocessing Personnel: Incidence and Reporting Practices in China

**DOI:** 10.1016/j.identj.2025.100944

**Published:** 2025-08-05

**Authors:** Feiruo Hong, Jiang Zeng, Junying Ma, Xiaoyan Wang, Xuefen Yu

**Affiliations:** a School of Medicine, Zhejiang University, Hangzhou, China; b Stomatology Hospital, School of Stomatology, Zhejiang University School of Medicine, Zhejiang Provincial Clinical Research Center for Oral Diseases, Key Laboratory of Oral Biomedical Research of Zhejiang Province, Cancer Center of Zhejiang University, Hangzhou, China

**Keywords:** Needlestick and sharp injury, Dental, Instrument, Blood-borne infection, Occupational safety

## Abstract

**Introduction and Aims:**

This study aimed to determine needlestick and sharps injuries (NSIs) incidence, identify associated risk factors, and describe reporting practices among dental instrument reprocessing personnel (DIRP) in China.

**Methods:**

A nationwide cross-sectional study utilized stratified multistage random sampling across seven Chinese regions. Validated questionnaires were administered to 1942 DIRP from 130 dental facilities, collecting data on demographics, occupational conditions, NSI history, and reporting. Multivariate logistic regression identified independent NSI predictors.

**Results:**

The one-year NSI period incidence was 21.7% (n = 421/1942), with 28.5% of these individuals experiencing multiple injuries. Daily instrument handling volume demonstrated a dose-response relationship with NSI risk (aOR = 5.73, 95% CI: 3.17-10.34, for >1000 vs. ≤100 instruments). The NSI reporting rate was 87.2%. Primary barriers included demanding work schedules (51.9%) and perceived low exposure risk (40.7%). Post-training assessment was significantly associated with a reduced NSI risk (aOR = 0.65, 95% CI: 0.51-0.83). Both receiving NSI prevention training (aOR = 3.11) and undergoing post-training assessment (aOR = 2.47) significantly increased NSI reporting.

**Conclusion:**

NSI prevalence among Chinese DIRP is substantial, strongly associated with instrument workload and work experience, and influenced by training assessment. Targeted interventions focusing on workload management, assessed training, and streamlined reporting are essential.

**Clinical Relevance:**

These findings highlight critical areas for improving DIRP safety. Implementing evidence-based workload limits, ensuring training includes competency assessment, and simplifying NSI reporting can reduce preventable injuries and enhance occupational health in dental settings globally.

## Introduction

Needlestick and sharp injuries (NSIs) are a pervasive occupational hazard for healthcare workers (HCWs) globally, including dental professionals. With an estimated three million percutaneous exposures occurring annually worldwide,^1^ NSIs pose significant risks of bloodborne pathogen transmission, including hepatitis B virus (HBV), hepatitis C virus (HCV), and human immunodeficiency virus (HIV), thereby compromising HCW safety and potentially impacting patient care quality.^2^ Beyond infectious risks, NSIs can cause substantial psychological distress, career disruption, and considerable healthcare costs,^3^, ^4^, ^5^ with many HCWs experiencing recurrent injuries that compound these adverse outcomes.^6^

The dental setting presents unique challenges regarding sharp safety due to the routine use of numerous sharp instruments such as endodontic files, burs, and probes, necessitating careful risk management, particularly concerning post-treatment instrument handling.^7^ While NSI risks among dentists and dental students have been extensively investigated,^8^^,^^9^ dental instrument reprocessing personnel (DIRP), those responsible for the critical initial stages of instrument reprocessing, remain comparatively understudied despite their distinctive occupational exposures.

In China, the dental healthcare sector has experienced rapid expansion, evidenced by a 51% increase in specialized hospitals for dental and oral medicine between 2015 and 2021.^10^ This growth has proportionally enlarged the dental workforce, including DIRP, who often operate under considerable time pressures and may possess limited specialized training in instrument reprocessing and hazard awareness.^11^ Unlike clinical HCWs whose primary sharps contact occurs during direct patient care, DIRP predominantly encounter instruments post-use. At this stage, blood and tissue residues can be dried and less visible, potentially diminishing situational awareness and thereby elevating injury risk.^12^ The intrinsic design of many dental instruments further elevates the potential for accidental punctures during sorting and manual cleaning.^13^ This vulnerability is potentially exacerbated by factors such as improper initial sharps disposal by clinicians,^14^ limited awareness or adherence to prevention protocol among DIRP themselves.^15^

Despite the established significance of NSIs, underreporting remains a persistent challenge in healthcare environments. Among dental support staff, such as dental assistants, underreporting rates can reach approximately 70%, often attributed to perceptions of low exposure risk, time constraints, and cumbersome reporting mechanisms.^16^ This widespread underreporting critically hinders the accurate assessment of the occupational injury burden and impedes the development of effective, targeted preventive strategies.^17^

Accordingly, this study aimed to (1) determine the incidence of NSI among DIRP in China, (2) identify associated demographic, professional, and organizational risk factors, and (3) assess NSI reporting behaviors and perceived barriers to reporting among this workforce.

## Methods

### Study design and population

A cross-sectional study was conducted between January and March 2024, targeting dental instrument reprocessing personnel (DIRP) across mainland China. For the purpose of this study, DIRP were defined as healthcare workers primarily responsible for the initial handling of contaminated dental instruments, as outlined in the Chinese National Regulation for Disinfection and Serialization Technique of Dental Instruments (WS 506-2016).^18^ Their roles include the retrieval of contaminated instruments from clinical areas, sorting instruments by type and complexity, and performing preliminary decontamination and initial cleaning. In essence, these personnel are on the frontline of the instrument reprocessing cycle, consistently handling contaminated items before they undergo automated washing or sterilization. Personnel whose roles were exclusively confined to disinfection processes or dedicated sterilization room operations were excluded.

### Sampling strategy and sample size

**Fig. 1:**
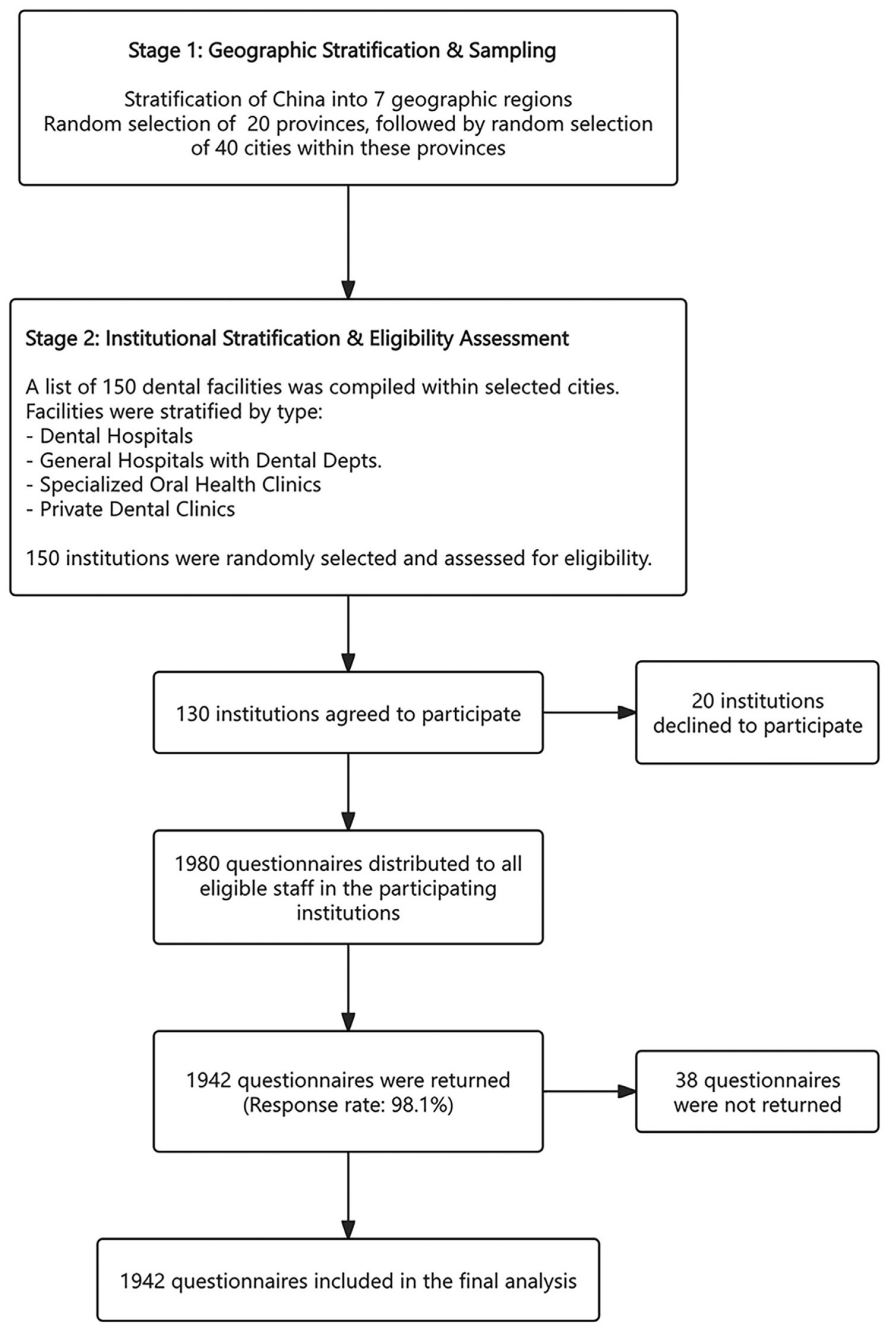
Flow diagram illustrating the stratified, multistage random sampling strategy and the participant selection process.

A stratified, multistage random sampling methodology was employed. The detailed process of participant recruitment and sample selection is illustrated in the flow diagram in Figure 1. Initially, China was stratified into seven geographic regions: East, Central, North, Northeast, Southwest, Northwest, and South. Within each region, provinces were randomly selected, followed by the random selection of cities within these chosen provinces. Subsequently, dental facilities within each selected city were stratified by type, including dental hospitals, general hospitals with dental departments, specialized oral health clinics, and small-scale private dental clinics.

The required sample size was calculated using Cochran's formula,^19^ a desired precision of ± 2%, and a 95% confidence level. Due to a lack of published data for this specific occupational group, an assumed NSI prevalence of 25% was used, a figure derived from a preliminary pilot study we conducted at a single dental hospital (n = 60). This yielded a minimum required sample of 1,800 DIRP. Accounting for a potential 10% non-response rate, the target sample size was adjusted to 1980 participants.

### Instruments and variables

Two purpose-developed, self-administered questionnaires were utilized: the “Supervisor Questionnaire” and the “DIRP Questionnaire” (see Supplementary Material A). The content validity of both instruments was established through review by an expert panel comprising seven specialists in infection control, occupational health, and dental practice. All items achieved a content validity index (CVI) exceeding 0.80. A pilot study with 30 participants, including 20 DIRP and 10 supervisors, was conducted to evaluate the feasibility and clarity of the questionnaires. Minor revisions were implemented based on feedback from this pilot phase, primarily involving simplification of technical terminology and reordering of questions to enhance flow and reduce ambiguity.

The “Supervisor Questionnaire” collected facility-level information, including geographical location, facility type, ownership structure, operational capacity, and management practices related to post-treatment instrument handling. The “DIRP Questionnaire” gathered individual-level data, including demographics, occupational characteristics, NSIs history, training received, and reporting behaviors. For participants reporting NSIs, additional questions assessed injury frequency (single vs. multiple incidents), characteristics, causes, and reporting practices.

### Data collection

Data were collected anonymously using an electronic questionnaire administered via Wenjuanxing (Questionnaire Star), a widely used online survey platform in China. The electronic format incorporated mandatory fields to prevent incomplete submissions and embedded logic checks to identify and prompt correction of inconsistent responses. Participation was voluntary, and electronic informed consent was obtained from all respondents before they could proceed with the survey. Logic verification checks were implemented throughout the data collection period to ensure data integrity and authenticity.

The study protocol received ethical approval from the Institutional Ethical Committee of Zhejiang University School of Stomatology (Approval No.002).

### Statistical analysis

Descriptive statistics were employed to summarize facility and participant characteristics, with categorical variables presented as frequencies and percentages. Bivariate associations between participant characteristics and NSI outcomes were examined using chi-square tests.

To identify independent predictors of NSI, three separate multivariate logistic regression models were developed. The outcomes of these models were: (1) occurrence of any NSI in the past 12 months; (2) reporting of the NSI among those who sustained an injury; and (3) experiencing multiple NSIs (≥ 2 incidents) among those with at least one NSI. Variables demonstrating a p-value < .25 in univariate analyses were considered for inclusion in the multivariate models, a threshold chosen to minimize the premature exclusion of potentially relevant factors in this exploratory analysis. All models were adjusted for the a priori selected potential confounders of age, gender, and work experience. The forced entry method was utilized for variable inclusion in the final models to ensure all preselected variables and confounders were assessed.

Model adequacy was evaluated using the Hosmer-Lemeshow test (*P > .*05 indicated adequate fit). Multicollinearity among independent variables was assessed by examining variance inflation factors (VIFs), with VIF <10 considered acceptable. Results from the logistic regression analyses are reported as adjusted odds ratios (aORs) with their 95% confidence intervals (CIs). A p-value ≤ .05 was considered indicative of statistical significance. All statistical analyses were performed using SPSS version 26.0 (IBM Corp., Armonk, NY, USA).

## Results

### Participant recruitment and facility characteristics

Of the 1980 DIRP invited to participate, 1,942 individuals from 130 dental facilities completed the survey, yielding a response rate of 98.1%.

The characteristics of the participating dental facilities are summarized in Table 1. A majority of facilities were geographically located in Eastern China (66.2%), followed by Central China (14.6%) and other regions. Dental hospitals represented the predominant facility type (70.0%). Most institutions operated under public ownership (66.2%). Facility capacity, indicated by the number of dental chairs, varied: 22.3% reported ≤ 10 chairs, 30.0% had 11-20 chairs, 27.7% had 21-50 chairs, and 20.0% possessed > 50 chairs. Regarding instrument processing practices, dental nurses were most commonly designated as the primary instrument handlers (88.5%). Instrument pre-treatment was predominantly conducted in central processing areas (47.7%), followed by dedicated serialization rooms (27.7%) and chairside processing (24.6%).

**Table 1:** Characteristics of participating dental facilities (N = 130).

Characteristics	*n* (%)
***Geographic location***	
Eastern China	86 (66.2)
Central China	18 (13.9)
Northern China	8 (6.1)
Other regions*	18 (13.8)
***Facility type***	
Dental hospital	91 (70.0)
General hospital (dental department)	18 (13.8)
Oral health clinic	16 (12.3)
Private dental practice	5 (3.9)
***Ownership***	
Public	86 (66.2)
Private	44 (33.8)
***Facility size (number of dental chairs)***	
≤10	29 (22.3)
11-20	39 (30.0)
21-50	36 (27.7)
> 50	26 (20.0)
***Primary instrument handlers***	
Dental nurse	115 (88.5)
Other personnel†	15 (11.5)
***Instrument pre-treatment location***	
Central processing area	62 (47.7)
Dedicated sterilization room	36 (27.7)
Chairside processing	32 (24.6)

⁎ Other regions include Northeastern, Southwestern, Northwestern, and Southern China.

† Other personnel include dental assistants (5.4%), dentists (4.6%), and interns/residents (1.5%).

### Participant characteristics

The demographic and occupational characteristics of the 1942 DIRP participants are detailed in Table 2, alongside the distribution of NSI incidence. The study cohort was overwhelmingly female (97.8%), with the largest age group being 20-29 years (62.6%). Dental nurses constituted the majority of professional roles (91.6%). Work experience varied, with 26.2% having 2-4.9 years, 25.1% having 5-10 years, and 19.4% having less than one year of experience. Daily instrument handling volumes also exhibited wide variation, with the majority (71.5%) processing ≤ 100 pieces of instruments daily, while 3.1% reported handling > 1000 pieces daily. The majority of participants (89.4%) reported receiving NSI prevention training. However, regular assessment of this training (post-training evaluation of their knowledge or skills) was reported by 64.8%.

**Table 2:** Participant characteristics and 12-month NSI incidence among dental instrument reprocessing personnel (N = 1942).

Characteristics	Total N (%)	NSI incidence N (%)
**Overall**	1942 (100)	421 (21.7)
***Gender***		χ² = 0.245, 1 df, *P = .*621
Male	43 (2.2)	8 (18.6)
Female	1899 (97.8)	413 (21.8)
***Age groups (years)***		χ² = 35.482, 3 df, ***P < .*001**
20-29	1215 (62.6)	216 (17.8)
30-39	592 (30.5)	157 (26.5)
40-49	107 (5.5)	36 (33.6)
≥50	28 (1.4)	12 (42.9)
***Occupational role***		χ² = 8.244, 3 df, ***P = .*041**
Dentist	62 (3.2)	12 (19.4)
Dental nurse	1778 (91.6)	380 (21.4)
Dental assistant	61 (3.1)	22 (36.1)
Resident and studen	41 (2.1)	7 (17.1)
***Professional rank***		χ² = 23.680, 3 df, ***P < .*001**
Junior	1226 (63.1)	241 (19.7)
Intermediate	478 (24.6)	140 (29.3)
Senior	19 (1.0)	5 (26.3)
Non-certified	219 (11.3)	35 (16.0)
***Education level***		χ² = 22.121, 4 df, ***P < .*001**
Junior high school or below	11 (0.6)	5 (45.5)
High school or vocational school	51 (2.6)	10 (19.6)
Associate degree	851 (43.8)	146 (17.2)
Bachelor's degree	1012 (52.1)	257 (25.4)
Master’s degree or above	17 (0.9)	3 (17.7)
***Work experience (years)***		χ² = 57.676, 4 df, ***P < .*001**
<1	256 (13.2)	30 (11.72)
1-1.9	374 (19.3)	46 (12.30)
2-4.9	509 (26.2)	116 (22.79)
5-10	457 (23.5)	125 (27.35)
>10	346 (17.8)	104 (30.06)
***Department***		χ² = 33.888, 7 df, ***P < .*001**
Comprehensive dentistry	573 (29.5)	139 (24.3)
Endodontics	172 (8.9)	44 (25.6)
Oral surgery	133 (6.8)	27 (20.3)
Prosthodontics	80 (4.1)	15 (18.8)
Orthodontics	200 (10.3)	23 (11.5)
Periodontics	102 (5.3)	24 (23.5)
Pediatric dentistry	229 (11.8)	29 (12.7)
Other specialties	453 (23.3)	120 (26.5)
***Facility type***		χ² = 9.077, 3 df, ***P = .*028**
Dental hospital	1532 (78.9)	334 (21.8)
General hospital (dental dept.)	141 (7.3)	41 (29.1)
Oral health clinic	246 (12.7)	40 (16.3)
Private dental practice	23 (1.2)	6 (26.1)
***Ownership***		χ² = 43.769, 1 df, ***P < .*001**
Public	950 (48.9)	266 (28.0)
Private	992 (51.1)	155 (15.6)
***Daily instrument handling volume (pieces)***		χ² = 100.999, 5 df, ***P < .*001**
≤100	1387 (71.5)	228 (16.4)
101-200	299 (15.4)	86 (28.8)
201-400	126 (6.5)	44 (34.9)
401-600	41 (2.1)	17 (41.5)
601-1000	28 (1.4)	14 (50.0)
>1000	61 (3.1)	32 (52.5)
***Received NSI prevention training***		χ² = 0.078, 1 df, *P = .*780
Yes	1737 (89.4)	375 (21.6)
No	205 (10.6)	46 (22.4)
***Underwent post-training assessment***		χ² = 19.926, 1 df, ***P < .*001**
Yes	1258 (64.8)	234 (18.60)
No	684 (35.2)	187 (27.4)

NSI, needlestick and sharp injury.

P-values calculated using chi-square test.

### NSI occurrence and associated risk factors

The overall one-year period prevalence of NSIs was 21.7% (n = 421/1942). Several demographic and occupational factors were significantly associated with NSI risk (Table 2, Table 3, Model 1).

**Table 3:** Multivariate logistic regression models for factors associated with NSIs occurrence, reporting, and multiple incidents.

Variable	Adjusted OR (aOR)	SE	95% CI	*P*-Value
Model 1. Outcome: NSI occurrence in past 12 months (N = 1942) Log-likelihood = 1846.970, χ^2^ = 183.628, *P < .*001, Hosmer-Lemeshow *P = .*395				
Work experience (years)				
<1	1.00 (ref)			
1-1.9	0.96	0.26	0.58-1.60	.887
2-4.9	1.85	0.24	1.16-2.96	**.010**
5-10	1.86	0.25	1.13-3.06	**.015**
>10	1.42	0.30	0.79-2.56	.241
**Department**				
Comprehensive dentistry	1.00 (ref)			
Endodontics	0.96	0.22	0.62-1.48	.838
Oral surgery	0.63	0.25	0.38-1.04	.068
Prosthodontics	0.62	0.32	0.33-1.16	.131
Orthodontics	0.42	0.25	0.25-0.68	**.001**
Periodontics	1.00	0.27	0.59-1.70	.992
Pediatric dentistry	0.50	0.23	0.32-0.79	**.003**
Other specialties	0.71	0.17	0.51-0.99	**.048**
**Daily instrument handling volume (pieces)**				
≤100	1.00 (ref)			
101-200	1.86	0.16	1.37-2.52	**<.001**
201-400	2.20	0.22	1.43-3.38	**<.001**
401-600	3.33	0.35	1.69-6.57	**.001**
601-1000	4.50	0.42	1.99-10.17	**<.001**
>1000	5.73	0.30	3.17-10.34	**<.001**
**Post-training assessment**				
No	1.00 (ref)			
Yes	0.65	0.12	0.51-0.83	**.001**
**Model 2. Outcome: NSIs reporting (N = 421)** **Log-likelihood = 291.691, χ^2^ = 30.860, *P = .*001, Hosmer-Lemeshow *P = .*571**				
**Received NSI prevention training**				
No	1.00 (ref)			
Yes	3.11	0.39	1.45-6.66	**.003**
**Post-training assessment**				
No	1.00 (ref)			
Yes	2.47	0.34	1.27-4.82	**.008**
**Model 3. Outcome: Multiple NSIs (vs. single, N = 421)** **Log-likelihood = 500.429, χ^2^ = 44.286, *P = .*003, Hosmer-Lemeshow *P = .*707**				
**Daily instrument handling volume**				
≤100	1.00 (ref)			
101-200	1.73	0.30	0.97-3.09	.065
201-400	1.23	0.39	0.57-2.67	.594
401-600	2.30	0.57	0.76-7.01	.143
600-1000	1.19	0.67	0.32-4.42	.793
>1000	3.33	0.43	1.42-7.80	**.006**

NSI, needlestick and sharps injury; OR, odds ratio; aOR, adjusted odds ratio; SE, standard error; CI, confidence interval.

All models were adjusted for age, gender, and work experience (unless work experience was the variable of interest in the model). Variables were included in multivariate models if *P < .*25 in univariate analysis or of theoretical importance.

A significant association was observed between age and NSI incidence (χ² = 35.482, *P < .*001), with rates increasing from 17.8% in the 20-29 years age group to 42.9% in those ≥ 50 years. Conversely, professional rank and education level demonstrated inverse relationships with NSI risk. Work experience exhibited a U-shaped association with NSI risk (χ²=57.676, *P < .*001). The lowest incidence was among those with < 1 year of experience (11.7%), rising to peak rates among those with 5-10 years (27.4%) and > 10 years of experience (30.1%). This non-linear pattern was maintained in the multivariate logistic regression analysis. Compared to the < 1 year reference group, the adjusted odds ratios (aORs) for NSI were 1.85 (95% CI: 1.16-2.96, *P = .*010) for DIRP with 2-4.9 years of experience and 1.86 (95% CI: 1.13-3.06, *P = .*015) for those with 5-10 years of experience.

Significant variations in NSI rates were observed across dental departments (χ²=33.888, *P < .*001). The highest rates were reported in endodontics (25.6%) and comprehensive dentistry (24.3%), while orthodontics (11.5%) and pediatric dentistry (12.7%) had significantly lower rates. After adjusting for confounders, working in orthodontics (aOR = 0.42, 95% CI: 0.25-0.68, *P = .*001) and pediatric dentistry (aOR = 0.50, 95% CI: 0.32-0.79, *P = .*003) remained significantly associated with lower odds of NSI compared to comprehensive dentistry.

The most prominent predictor of NSI risk was the daily volume of instruments handled, which demonstrated a strong dose-response relationship in both univariate (χ²=100.999, *P < .*001) and multivariate analyses. Personnel handling > 1000 instrument pieces daily had a 5.73-fold increased adjusted odds of experiencing an NSI (95% CI: 3.17-10.34, *P < .*001) compared to those handling ≤ 100 pieces daily. While having received NSI prevention training alone was not significantly protective, undergoing post-training assessment of knowledge or skills was associated with a reduced risk of NSI (aOR=0.65, 95% CI: 0.51-0.83, *P = .*001).

### NSI reporting behavior

Among the 421 participants who sustained an NSI in the preceding year, 367 (87.2%) reported their injuries. Reporting rates varied significantly with several characteristics (Supplementary Table S1). Both receiving NSI prevention training (χ²=22.265, *P < .*001) and undergoing post-training assessment (χ²=12.420, *P < .*001) were significantly associated with higher reporting rates. Specifically, DIRP, who received training, reported 89.9% of incidents, compared to 65.2% among those who had not received training.

Multivariate analysis of reporting behavior (Table 3, Model 2) confirmed these associations. Receiving NSI training (aOR = 3.11, 95% CI: 1.45-6.66, *P = .*003) and undergoing post-training assessment (aOR = 2.47, 95% CI: 1.27-4.82, *P = .*008) significantly increased the likelihood of NSI reporting. The principal reasons cited for non-reporting included being too busy (51.9%), perceiving a low risk of exposure risk (40.7%), and complicated reporting procedures (38.9%) (Table 4).

**Table 4:** Characteristics of NSIs, reporting, and training among participants with injury experience (N = 421).

Characteristics	N	%
***NSI frequency in past 12 months***		
1 time	274	65.1
2-3 times	127	30.2
>3 times	20	4.7
***Practices during instrument handling at time of injury***		
Using hands, wearing gloves	70	16.6
Using tools, wearing gloves	349	82.9
Using tools, not wearing gloves	2	0.5
Instruments causing NSIs (Total NSI events = 680)*		
Endodontic files	171	25.2
Dental probe	159	23.4
Syringe	138	20.3
Dental burs	94	13.8
Other sharp instruments	94	13.8
Scalpel blade	24	3.5
***Bleeding at injury site***		
No	297	70.5
Yes	124	29.5
***Immediate actions post-exposure***		
Ignored, continued working	1	0.2
Washed and sterilized injury site	11	2.6
Squeezed blood and sterilized injury site	12	2.9
Adhered to institutional post-exposure protocol	397	94.3
***NSI reporting (421)***		
Reported	367	87.2
Not reported	54	12.8
Reason for not reporting (n=54 respondents)*		
Too busy	28	51.9
Perceived low exposure risk	22	40.7
Complicated reporting process	21	38.9
Unaware of NSI reporting procedures	4	7.4
***NSI prevention training format received***		
Offline training	286	67.9
Online training	63	15.0
No training received	46	10.9
Self-study with materials	10	2.4
Mixed format (online and offline)	16	3.8
NSI prevention training content covered*		
Injury site treatment/First aid	326	77.4
NSI concepts and transmission risks	258	61.3
NSI reporting procedures	253	60.1
NSI prevention measures	236	56.1
Primary NSI training provider*		
Infection control department	186	44.2
Head nurse	150	35.6
Colleague	83	19.8
Department director	2	0.5
NSI training assessment method*		
Theoretical examination	192	45.6
Practical operation evaluation	153	36.3
Observation of practice	99	23.5
Others methods	26	6.2

NSI, needlestick and sharps injury.

⁎ Multiple responses permitted; percentages calculated based on the number of respondents (n = 421 for training content/provider/assessment; n = 54 for reasons for non-reporting), or total NSI events (n = 680 for instruments causing NSIs). Therefore, percentages may sum to >100%.

### Multiple NSI events

Of the 421 DIRP who experienced an NSI, 147 (34.9%) reported experiencing multiple NSI incidents within the past 12 months. Univariate analyses indicated that occupational role (χ² = 8.379, *P = .*039), professional rank (χ² = 21.335, *P < .*001), and education level (χ² = 21.490, *P < .*001) were significantly associated with the occurrence of multiple NSIs. However, in the multivariate analysis of risk factors for multiple NSIs (Table 3, Model 3), only daily instrument handling volume emerged as a significant independent predictor. DIRP handling > 1000 instrument pieces daily had 3.33 times higher adjusted odds of experiencing multiple NSIs (95% CI: 1.42-7.80, *P = .*006) compared to those handling ≤ 100 pieces daily.

### Characteristics of reported NSIs

As shown in Table 4, among participants who experienced an NSI, the majority of injuries (82.9%) occurred during instrument handling while using tools with gloves. The most frequently implicated instruments in NSIs were endodontic files (25.2%), dental probes (23.4%), and syringes (20.3%). Critically, 29.5% of these injuries resulted in bleeding, indicating a direct breach of the skin barrier. A high proportion (94.3%) of injured personnel reported adhering to post-exposure protocols.

Regarding NSI prevention training among those who sustained an injury, 67.9% had received offline training, while 10.9% reported receiving no training at all. The most common topics covered in training included immediate care of the injury site (77.4%), fundamental concepts of NSIs (61.3%), and NSI reporting procedures (60.1%). Training was primarily delivered by infection control departments (44.2%) or head nurses (35.6%). The predominant methods for assessing training effectiveness were theoretical examinations (45.6%) and observation of practical skills (36.3%).

## Discussion

This large-scale investigation offers crucial insights into the epidemiology of needlestick and sharp injuries (NSIs) among dental instrument reprocessing personnel (DIRP) in China, a group often overlooked in occupational health research within dental settings. The observed 12-month NSI incidence of 21.7% highlights a significant occupational burden. This finding falls within the spectrum of NSI prevalence reported globally among healthcare workers in developing countries, which ranges from 19.9% to 54.0% annually.^20^ While direct comparisons are challenging due to methodological variations, our rate is noteworthy when considering specific dental support staff. A recent systematic review focusing on dental assistants reported a pooled NSI prevalence of 44%,^16^ suggesting that DIRP, who perform similar handling tasks with contaminated instruments, also face substantial risks, though with variations influenced by their specific roles and work environments. Our study makes a unique contribution through its dedicated focus on this DIRP population at a national scale in China.

Beyond the high incidence of NSIs, one of our striking findings is that nearly one-third of these injuries resulted in bleeding. This figure is highly concerning, as visible bleeding is a definitive indicator of a percutaneous injury that breaches the skin barrier. Such exposures dramatically elevate the potential for transmission of blood-borne pathogens like HBV, HCV, and HIV.^21^ This single statistic confirms that a substantial portion of these incidents are not minor scratches but are, in fact, high-risk events with serious clinical implications. This underscores the critical importance of a robust system for comprehensive prevention and immediate post-exposure management.

The multivariate analysis identified several significant predictors of NSI occurrence. Notably, daily instrument handling volume emerged as a powerful, dose-dependent predictor of NSI risk. The substantially increased odds of injury, including multiple NSI events, among personnel processing higher instrument volumes underscore that workload is a critical risk factor. This finding resonates with research from broader healthcare settings where workload is consistently identified as an NSI determinant^20^ and aligns with established ergonomic principles where increased task repetition elevates injury likelihood.^22^^,^^23^ The robust association strongly advocates for implementing workload management strategies, a point also emphasized by Imran et al.,^7^ who highlight the sharp-risk environment in both clinical and decontamination units in dentistry. The powerful association between workload and NSI risk must be interpreted within the context of China's national infection control landscape. While China has established comprehensive national guidelines for the handling of contaminated dental instruments, such as the Chinese National Regulation for Disinfection and Serialization Technique of Dental Instrument (WS 506-2016),^18^ our findings suggest a significant gap between policy and practice. The high workloads likely act as a major barrier to the consistent application of these guidelines at the institutional level. When personnel are under pressure to process large volumes of instruments, adherence to safety protocols may be compromised, leading to shortcuts that elevate injury risk.^24^ This indicates that the mere existence of institutional processes is insufficient; effective implementation requires adequate staffing and workload management to create a feasible environment for safe practice.

Our findings revealed significant departmental variations in NSI risk, with personnel in orthodontics and pediatric dentistry exhibiting lower risk compared to comprehensive dentistry. This suggests that the nature of procedures and instrument characteristics inherent to different dental specialties may independently influence injury risk. The lower NSI risk in orthodontics and pediatric dentistry likely reflects less invasive procedures or instrument designs with fewer sharp features. Conversely, the greater instrument diversity and procedural complexity in comprehensive dentistry could contribute to increased accidental injuries. These specialty-specific findings imply that safety interventions should be tailored to departmental contexts rather than uniformly applied, aligning with recommendations from the International Labor Organization's guidelines on healthcare worker safety.^25^

Challenging conventional assumptions, our analysis uncovered a non-linear relationship between work experience and NSI risk. DIRP with intermediate experience levels (2-10 years) demonstrated higher odds of injury compared to novices. This U-shaped pattern contrasts with a previous investigation of Saudi Arabian dentists^26^ but aligns with observations in other settings where experienced personnel might develop risk normalization.^27^^,^^28^ Interestingly, Iwamatsu-Kobayashi et al. found a high proportion of NSIs in dental healthcare workers with less than five years of experience in Japan, potentially reflecting the initial learning curve and one arm of such a U-shaped distribution.^29^ Our finding suggests that for DIRP, the period beyond initial acclimatization but before extensive long-term experience might represent a window of increased vulnerability. This highlights the need for cyclical safety interventions, such as regular refresher training, rather than solely front-loaded training.^30^

The distinction between training provision and training effectiveness emerged as another critical insight. The finding that training alone did not independently predict a reduction in NSI occurrence is consistent with the finding of a study of Chinese dental nurses.^31^ However, undergoing post-training assessment was significantly associated with lower NSI risk. This emphasizes that the quality and evaluation of training are important for translating knowledge into safe practice, rather than its simple delivery.^32^ Competency-based safety programs, which emphasize active learning and skill consolidation through assessment, appear to be a more efficient interventional strategy.

Despite a relatively high overall NSI reporting rate in our study, persistent underreporting remains a concern. Key self-reported barriers, including demanding work schedules and perceived low exposure risk, align with reasons for non-reporting found elsewhere, such as considering the injury minor.^33^ The perception of low risk is particularly alarming when contrasted with our finding that nearly one-third of all reported injuries involved bleeding. This disparity points to a persistent knowledge gap or a normalization of risk among staff, a concern also raised by Xin et al. regarding awareness among dental personnel.^34^ The complexity of reporting procedures further deters reporting, a systemic issue that needs addressing. A critical implication of underreporting relates to the management of post-exposure prophylaxis (PEP). Effective PEP is time-sensitive and serves as the last line of defense against serious infections like HIV, HBV, and HCV.^35^ The barriers to reporting create a dangerous situation where affected personnel may not undergo the necessary risk assessment or receive timely PEP. Therefore, interventions must not only aim to reduce NSI incidence but also to dismantle reporting barriers by fostering a non-punitive culture^36^ and streamlining post-injury protocols to ensure immediate and universal access to post-exposure care.

Interestingly, both receiving training and undergoing post-training assessment significantly increased the likelihood of injury reporting. This suggests that educational interventions may be more influential in shaping post-injury behavior than in primary NSI prevention itself. This is supported by Persoon et al.,^37^ who found that oral healthcare professionals with accurate knowledge of blood exposure accident protocols were more likely to report, although paradoxically also more likely to experience such accidents, highlighting the complex interplay of knowledge, behavior, and reporting. Our findings suggest that robust training and assessment not only aim to prevent incidents but also foster a better understanding of consequences and reporting importance.

Current NSI prevention training practices for DIRP, predominantly offline and guided by infection control departments, show room for improvement. While focusing on essential areas like wound management and reporting, a concerning proportion of injured DIRP received no training. The reliance on theoretical examinations as the primary assessment method may not adequately evaluate practical competencies.^38^ Future initiatives should diversify training delivery and assessment methods, incorporating practical evaluations and simulations to enhance efficacy. The high proportion of DIRP experiencing multiple NSIs further identifies a vulnerable subpopulation, for whom instrument handling volume was the sole significant predictor, reinforcing the critical need for workload management to prevent both initial and recurrent injuries. Beyond optimizing training and managing workload, technological advancements offer a promising avenue for fundamentally re-engineering the safety of this process. As highlighted in a recent review by AlSaiari et al.,^39^ bioengineering innovations hold significant potential to mitigate the inherent risks in dental infection control.

Specific instruments like endodontic files, dental probes, and syringes were commonly implicated in NSIs, reflecting the inherent risks associated with their sharp designs and handling during reprocessing.^40^^,^^41^ The observation that most incidents occurred despite glove use and tool assistance suggests that current basic protective measures, while generally implemented, are not completely effective or may suffer from inconsistent application. The high reported adherence to post-exposure protocols is positive, though potential reporting bias warrants cautious interpretation.

This study benefits from a large, nationally representative sample and robust multivariate modelling. Nevertheless, several limitations warrant consideration. First, the cross-sectional design precludes definitive causal inference.^42^ While we identified strong associations, we cannot definitively conclude a cause-and-effect relationship. Longitudinal studies would be beneficial to confirm these findings. Second, the self-reporting of NSI introduces the potential for recall bias and underestimation.^43^ It is plausible that minor injuries that did not result in significant bleeding or pain were forgotten, meaning our calculated incidence may represent a conservative estimate of the true occupational risk. Finally, our study did not collect specific data on the types of post-exposure prophylaxis received or audit the specific infection control practices within each participating institution. Future research integrating direct observation and institutional audits would provide a more complete picture of the interplay between policy, practice, and injury outcomes.

## Conclusions

This cross-sectional study provides significant evidence on the epidemiology of NSIs among DIRP in China, revealing a substantial 12-month period prevalence of 21.7% and identifying critical risk factors, most notably daily instrument handling volume, specific dental departments, work experience dynamics, and the pivotal role of post-training assessment. Future research should focus on evaluating the effectiveness of multifaceted strategies that address workload management, implement comprehensive training programs incorporating assessment components, and establish simplified NSI reporting systems to reduce the burden of these preventable injuries in dental healthcare settings.

## Ethics statement

The study was approved by the Institutional Ethical Committee of Zhejiang University School of Stomatology. Number of the approval 002 (January 11, 2024)

## Statement

During the preparation of this work the authors used Doubao in order to increasing the readability of the English language. After using this tool, the authors reviewed and edited the content as needed and take full responsibility for the content of the publication.

## Funding

This work was supported by the Medical Science and Technology Project of Zhejiang Province [grant number: 2023KY131].

## CRediT authorship contribution statement

**Feiruo Hong:** Methodology, Formal analysis, Writing – original draft. **Jiang Zeng:** Methodology, Formal analysis, Writing – review & editing. **Junying Ma:** Investigation, Data curation. **Xiaoyan Wang:** Investigation, Data curation. **Xuefen Yu:** Conceptualization, Methodology, Writing – review & editing, Supervision, Funding acquisition.

## Declaration of competing interest

The authors declare that they have no known competing financial interests or personal relationships that could have appeared to influence the work reported in this paper.

## Appendix Supplementary materials

Image, application 1

## Footnotes

Supplementary material associated with this article can be found in the online version at doi:10.1016/j.identj.2025.100944.
