# Supplementary material for: Needlestick and Sharp Injury Among Dental Instrument Reprocessing Personnel: Incidence and Reporting Practices in China
Source: Int Dent J. 2025 Aug 5;75(5):100944. doi: 10.1016/j.identj.2025.100944 (PMC12345279; doi:10.1016/j.identj.2025.100944)
Supplement: Supplementary file 1 [file mmc1.docx]

Supplementary A：

**Survey on the Current Status of Instrument Reprocessing Management in Dentistry (Supervisor Questionnaire)**

Dear Participant,

This survey aims to explore the current status of instrument reprocessing management in dentistry. Please answer the following questions truthfully. Your responses will be kept anonymous and used solely for research purposes. Thank you for your support and cooperation!

**Section I: Basic Information**

1. What type of medical institution do you work at?

- A. Dental hospital

- B. General hospital

- C. Oral health clinic

- D. Private dental practice

2. What is the nature of your medical institution?

- A. Public

- B. Private

3. What is your position?

- A. Chief Nursing Officer

- B. Head of Infection Control Department

- C. Head Nurse

- D. Nursing Team Leader

4. How many dental treatment chairs are there in your department/medical institution?

- A. 1-10

- B. 11-20

- C. 21-50

- D. 51-100

- E. Over 100

5. What is the daily outpatient volume in your medical institution/department?

- A. Less than 10 patients

- B. 11-50 patients

- C. 51-100 patients

- D. 101-200 patients

- E. 201-500 patients

- F. Over 500 patients

**Section II: Instrument Reprocessing Process**

1. Who usually handles the treatment tray after the treatment?

- A. The treating dentists

- B. The assisting nurse/assistant

- C. Nursing assistant

- D. Intern/resident/rotating trainee

2. Where are dental instruments processed?

- A. Chairside processing

- B. Central processing area

- C. Sterilization room

- D. Other: __________

**Section III: Management of Reprocessing Personnel**

1. Which reprocessing personnel in your unit have received specialized training in instrument reprocessing? (Multiple choices allowed)

- A. Dentists

- B. Nurses/Assistants

- C. Interns/Residents/Rotating Trainees

- D. Nursing assistants

2. What are the contents and methods of specialized instrument handling training?

- A. Brief introduction to the environment and scope of work

- B. Introduction to the characteristics and classification requirements of instruments

- C. Introduction to the characteristics, classification requirements, and sorting methods to avoid punctures

- D. Introduction to the characteristics, disposal methods, classification requirements, and sorting methods to avoid punctures, with demonstration and practice

3. Have instrument disposal personnel received training on occupational exposure to sharp injuries?

- A. Yes

- B. No

3.1 If training on occupational exposure to sharp injuries has been received, what methods were used?

- A. Lecture-based training methods (lecture, seminar)

- B. Practice-based training methods (demonstration and simulation)

- C. Technology-based training methods (audiovisual, online training, video training)

- D. Other: __________

3.2 How frequently is training on occupational exposure to sharp injuries conducted?

- A. Once after employment

- B. Once a year

- C. Twice a year

- D. Once a quarter

- E. Once a month

3.3 Is there an assessment test for occupational exposure training? (If yes, please answer the following questions)

- A. Yes

- B. No

3.3.1 Methods of assessment testing

- A. Theoretical assessment

- B. Practical assessment

- C. Observation assessment

- D. Other: __________

4. Is there a reporting system for occupational exposure to sharp injuries?

- A. Yes

- B. No

**Survey on the Current Status of Instrument Reprocessing in Dentistry (DIRP Questionnaire)**

Dear Participant,

This survey aims to explore the current status of instrument reprocessing by personnel in dentistry. Please answer the following questions truthfully. Your responses will be kept anonymous and used solely for research purposes. Thank you for your support and cooperation!

**Section I: Basic Information**

1. What type of medical institution do you work at?

- A. Dental hospital

- B. General hospital

- C. Oral health clinic

- D. Private dental practice

2. What is the nature of your medical institution?

- A. Public

- B. Private

3. What is your gender?

- A. Male

- B. Female

4. What is your age?

- A. 20-29 years old

- B. 30-39 years old

- C. 40-49 years old

- D. Over 50 years old

5. What is your occupation?

- A. Dentist

- B. Nurse

- C. Nursing assistant

- D. Other: __________

6. What is your professional title?

- A. Junior title

- B. Intermediate title

- C. Senior title

- D. None

7. What is your educational background?

- A. Junior high school or below

- B. High school / Vocational school

- C. Associate degree

- D. Bachelor's degree

- E. Graduate degree or above

8. How long have you been working at your current institution?

- A. Less than 1 year

- B. 1-1.9 years

- C. 2-4.9 years

- D. 5-10 years

- E. Over 10 years

9. Which department do you work in?

- A. Comprehensive department

- B. Endodontics

- C. Oral surgery

- D. Prosthodontics

- E. Orthodontics

- F. Periodontics

- G. Pediatric dentistry

- H. Other: __________

**Section II: Instrument reprocessing Process**

1. How do you sort small dental instruments?

- A. By hand with gloves

- B. With tweezers or other tools while wearing gloves

- C. With tweezers or other tools without gloves

1.1 Why do you sort instruments by hand?

- A. It is convenient and fast

- B. I have not considered the risk of puncture

- C. Using tweezers or other tools is troublesome and inefficient

2. How many instruments do you reprocess in a day?

- A. 1-100 pieces

- B. 101-200 pieces

- C. 201-400 pieces

- D. 401-600 pieces

- E. 601-1000 pieces

- F. Over 1000 pieces

3. Have you ever experienced a sharp injury while reprocessing instruments in the past year? (If yes, please answer the following questions)

- A. Yes

- B. No

3.1 In the past year, how many times have you experienced a sharp injury while reprocessing instruments?

- A. 1 time

- B. 2 times

- C. 3 times

- D. 4 times

- E. 5 times

- F. More than 6 times

3.2 What was the severity of your injury?

- A. Mild (superficial puncture; no bleeding or minimal bleeding)

- B. Moderate (skin puncture with bleeding)

- C. Severe (deep puncture with significant bleeding)

3.3 Which instruments caused your sharp injury? (Multiple choices allowed)

- A. Syringe needle

- B. Probe

- C. Blade

- D. Bur

- E. File

- F. Other sharp instruments

3.4 How did you handle the sharp injury?

- A. Did not treat it and continued sorting instruments

- B. Rinsed with water and disinfected the wound

- C. Squeezed the wound to expel a small amount of blood and disinfected the wound

- D. First squeezed the injured area to expel contaminated blood, then rinsed with water, dried, and disinfected

3.5 Did you report the sharp injury to the head nurse or other managers?

- A. Yes

- B. No

3.5.1 If you did not report the injury according to the standard procedure, what were the reasons? (Multiple choices allowed)

- A. Did not know the handling procedure

- B. Too busy to handle it

- C. The standard handling procedure is too cumbersome

- D. Did not think it was necessary to report

3.6 When you were injured by a contaminated instrument, were you worried about contracting an infectious disease?

- A. Very worried

- B. Somewhat worried

- C. Not worried

**Section III: Management of Reprocessing Personnel**

1. Are you aware of the standard procedure for handling sharp injuries?

- A. Yes

- B. No

2. Have you received training related to instrument sorting?

- A. Yes

- B. No

2.1 What methods of training have you received? (Multiple choices allowed)

- A. Offline training

- B. Self-study

- C. Online training

- D. Other

2.2 What topics were covered in your training? (Multiple choices allowed)

- A. Concept of sharp injury occupational exposure

- B. Methods to avoid sharp injuries

- C. Wound management after a sharp injury

- D. Reporting procedures after a sharp injury

3. Who provided your training?

- A. Head nurse

- B. Infection control department staff

- C. Department head

- D. Nurse

- E. Colleague

4. Does your unit/department conduct regular assessments? (If yes, please answer the following questions)

- A. Yes

- B. No

4.1 What types of assessments are conducted? (Multiple choices allowed)

- A. Theoretical assessment

- B. Practical assessment

- C. Observation assessment

- D. Other

**Table S1 Univariate Analysis of Factors Associated with NSI Reporting and Multiple Incidents**

| **Characteristic** | **Reported NSIs**  **N (%)** | **Multiple NSIs**  **N (%)** |
| --- | --- | --- |
| Total | 367(87.2) |  |
| Gender | χ²=2.476, 1 df, P=0.116 | χ²=0.779, 1 df, P=0.378 |
| Male | 5(62.5) | 4 (50.0) |
| Female | 362(87.7) | 143 (34.6) |
| Age groups, years | χ²=4.101, 3 df, P=0.251 | χ²=4.437, 3 df, P=0.218 |
| 20-29 | 185(85.7) | 78 (36.1) |
| 30-39 | 139(88.5) | 48 (30.6) |
| 40-49 | 34(94.4) | 14 (38.9) |
| ≥50 | 9(75.0) | 7 (58.3) |
| Occupational role | χ²=2.407, 3 df, P=0.492 | χ²=8.379, 3 df, P=0.039 |
| Dentist | 10(83.3) | 4 (33.3) |
| Dental nurse | 330(86.8) | 131 (34.5) |
| Dental assistant | 20(90.9) | 6 (27.3) |
| Resident and student | 7(100.0) | 6 (85.7) |
| Professional rank | χ²=2.255, 3 df, P=0.521 | χ²=21.335, 3 df, P<0.001 |
| Junior | 209(86.7) | 89 (60.5) |
| Intermediate | 121(86.4) | 35 (25.0) |
| Senior | 4(80.0) | 5 (100) |
| Non-certified | 33(94.3) | 18 (51.4) |
| Education level | χ²=1.653, 4 df, P=0.799 | χ²=21.490, 4 df, P<0.001 |
| Junior high school or below | 4(80.0) | 4 (80.0) |
| High school or vocational school | 8(80.0) | 5 (50.0) |
| Associate degree | 129(88.4) | 67 (45.9) |
| Bachelor's degree | 223(86.8) | 71 (27.6) |
| **Master’s degree or higher** | 3(100) | 0 (0) |
| Work experience, years | χ²=2.013, 4 df, P=0.733 | χ²=8.977, 4 df, P=0.062 |
| <1 | 24(80.0) | 16 (53.3) |
| 1-1.9 | 40(87.0) | 21 (45.7) |
| 2-4.9 | 100(86.2) | 37 (31.9) |
| 5-10 | 112(89.6) | 43 (34.4) |
| >10 | 91(87.5) | 30 (28.8) |
| Department | χ²=10.707, 7 df, P=0.152 | χ²=8.856, 7 df, P=0.263 |
| Comprehensive dentistry | 113(81.3) | 45 (32.4) |
| Endodontics | 38(86.4) | 14 (31.8) |
| Oral surgery | 24(88.9) | 6 (22.2) |
| Prosthodontics | 15(100.0) | 3 (20.0) |
| Orthodontics | 20(87.0) | 12 (52.2) |
| Periodontics | 21(87.5) | 8 (33.3) |
| Pediatric dentistry | 27(93.1) | 10 (34.5) |
| Other specialties | 109(90.8) | 49 (40.8) |
| Type of institution | χ²=3.195, 3 df, P=0.363 | χ²=6.154, 3 df, P=0.104 |
| Dental hospital | 294(88.0) | 116 (34.7) |
| General hospital | 32(78.0) | 14 (34.1) |
| Oral health clinic | 36(90.0) | 17 (42.5) |
| Private dental practice | 5(83.3) | 0 (0) |
| Ownership type | χ²=0.323, 1 df, P=0.570 | χ²=5.318, 1 df, P=0.021 |
| Public | 230(86.5) | 82 (30.8) |
| Private | 137(88.4) | 65 (41.9) |
| Daily instrument handling volume | χ²=4.852, 5 df, P=0.434 | χ²=6.830, 5 df, P=0.234 |
| ≤100 | 202(88.6) | 73 (32.0) |
| 101-200 | 74(86.0) | 31 (36.0) |
| 201-400 | 39(88.6) | 14 (31.8) |
| 401-600 | 13(76.5) | 8 (47.1) |
| 601-1000 | 10(71.4) | 4 (28.6) |
| >1000 | 29(90.6) | 17 (53.1) |
| Received NSIs training | χ²=22.265, 1 df, P<0.001 | χ²=0.403, 1 df, P=0.525 |
| Yes | 337(89.9) | 129 (34.4) |
| No | 30(65.2) | 18 (39.1) |
| Post-training assessment | χ²=12.420, 1 df, P<0.001 | χ²=0.004, 1 df, P=0.952 |
| Yes | 216(92.3) | 82 (35.0) |
| No | 151(80.7) | 65 (34.8) |
